# School district-wide renovations, indoor environmental quality, and illness absence

**DOI:** 10.1038/s41370-026-00903-5

**Published:** 2026-05-06

**Authors:** Ulla Haverinen-Shaughnessy, Samy Clinchard, Mark Hernandez, Wanda Phipatanakul, Richard Shaughnessy

**Affiliations:** 1 The University of Tulsa, Tulsa, OK USA; 2 University of Oulu, Oulu, Finland; 3 University of Colorado Boulder, Boulder, CO USA; 4 Boston Children’s Hospital and Harvard T.H. Chan School of Public Health, Boston, MA USA

**Keywords:** IEQ, gastrointestinal, K-12 schools, respiratory, surface biocontamination, thermal conditions, ventilation

## Abstract

**Background:**

The physical school environment is a key factor influencing illness-related absences, particularly those caused by infectious diseases transmitted through air and surfaces. Previous studies, often underpowered, have been unable to conclusively quantify the impacts of improved classroom indoor environmental quality (IEQ) on illness-related absences, while also accounting for sociodemographic factors, such as gender and ethnicity.

**Objective:**

This study evaluates the impacts of district-wide renovations on student absences due to specific illness types, based on exceptional district data on daily absenteeism and socioeconomic factors, covering 1217 school days and 45,428 students.

**Methods:**

Between 2016 and 2021, over 45 schools in a district located in a mountain west region of United States underwent comprehensive renovations, which included upgrading heating, ventilation, and air conditioning systems. At the same time, cleaning protocols were developed to reduce surface contamination. Data from pre- and post-renovation monitoring included classroom temperature, relative humidity, and surface biocontamination levels. Carbon dioxide levels were used to estimate ventilation rates, while temperature and relative humidity were used to estimate absolute humidity.

**Results:**

Independent associations between illness-specific absences and school renovations, along with several IEQ parameters, were quantified. Based on the results, over one-third of absences due to respiratory illnesses could be reduced by keeping school facilities up to date and adhering to recommended standards for ventilation. Increased frequency of cleaning could help to reduce absences due to gastrointestinal illnesses. Additional reductions could be achieved by maintaining higher humidity and cooler temperatures in classrooms.

**Significance:**

These findings highlight the importance of maintaining good IEQ in schools, resulting in fewer absences from infectious diseases.

**Impact:**

The study is based on a large-scale natural experiment, incorporating daily student-level absence data specifying illness types and background information for all students in the district, collected over seven school years - the longest follow-up period to date, to our knowledge. These data, combined with state-of-the-art IEQ monitoring conducted before and after the renovation of 45 schools, provides superior statistical power to quantify the impacts of renovations and how IEQ parameters may partially explain their effects on illness-related absences. The findings can inform policies at the school, district, and national levels, guiding renovations, maintenance and operational practices aimed at reducing airborne pathogens and surface contamination, and reducing illness-related absenteeism in schools.

## Introduction

Health conditions associated with school absenteeism include infectious diseases, fractures, asthma, diabetes, pain, seizures, poor oral health, obesity, and mental health conditions [1]. Other factors include poverty, unstable housing conditions, and ethnic minority status.

Intuitively, the role of the schools’ physical environment in student absenteeism is significant but not yet fully understood. Students spend a large portion of their day indoors, where building characteristics, such as ventilation, thermal conditions, moisture control, and surface materials influence exposure to airborne and surface-associated pathogens, allergens, and other environmental stressors [2]. These exposures can affect susceptibility to infectious and respiratory illnesses, which are among the leading causes of school absence. Better understanding of these relationships enables the development and implementation of evidence-based tools, policies, programs, and interventions, which can help to ensure safe and healthy school environments [3]. Suggested interventions to improve school attendance include strategies to prevent infection transmission [4, 5]. While previous studies have focused on classroom IEQ (e.g., ventilation and surface cleanliness), they have often been cross-sectional and lacked information on the specific types of illnesses causing absences. Furthermore, outcomes are typically assessed only at school level.

Since the COVID-19 pandemic, several review articles have been published on ventilation in schools and infectious diseases, suggesting the need for new health-based ventilation approaches in classrooms [6, 7]. In addition, thermal conditions in classrooms is an important factor impacting both student health and performance[8]. Thermal conditions are affected by both temperature and humidity, where the latter has also been associated with the transmission, occurrence, and prevalence of infectious diseases but has not been commonly studied in relation to illness-related absenteeism [9, 10].

Many pathogens can survive on surfaces for days to months [11, 12]. Therefore, regular preventive surface cleaning and disinfection have been recommended to reduce the risk of indirect transmission. However, there is a lack of evidence related to how effective surface cleaning is in reducing absenteeism.

Previous cross-sectional studies in the southwest and south-central U.S. linked classroom ventilation rate, temperature, and surface cleanliness to student health and performance [13]. In spring 2020, Shaughnessy et al. conducted a 10-week intervention with thirty-four schools (15,814 students) randomly assigned to implement either enhanced or standard cleaning [14]. Based on the results, enhanced cleaning significantly reduced biocontamination on desktops, which was associated with gastrointestinal illness-related absences.

The current study is based on a unique opportunity to assess the co-benefits of significantly improving IEQ in schools, following a $600 million bond program in a school district within the Mountain West region of the U.S. This initiative funded upgrades, including modern heating, ventilation, and air conditioning (HVAC) systems. We monitored IEQ parameters and collected extensive pre- and post-renovation data to assess the effects of improved school environment on specific types of illness-related absences among students.

## Materials and Methods

In 2016–2017, baseline data were collected from 50 schools. Over the following years, 45 schools were renovated in three phases. First set of 15 schools were renovated between March 2015–December 2017 (Phase 1), following schools were renovated between May 2016 – March 2020 (Phase 2), and last set of schools between April 2019–July 2021 (Phase 3). The average duration was 21 (range 12–72) months. Other background information about the schools is presented elsewhere [14, 15].

Through renovations, the district aimed to improve both energy efficiency and IEQ, including meeting current standards for heating, ventilation, air conditioning, and cleanliness [16, 17]. During and after the renovations, the school management received feedback and adjusted ventilation and cleaning procedures accordingly.

The data collection was repeated in three phases following the renovation. The data collection approach is consistent with prior studies [17] and aligns with harmonized monitoring protocols develope to identify and address environmental risk factors in schools [18]. Data collection began with comprehensive walkthroughs of each school, using pre-designed checklists and simple measurements (i.e., ‘spot checks’) in classrooms. The walkthrough data were used to carefully select a representative sample of 3-6 classrooms from each school for continuous monitoring. The selected classrooms represented different HVAC zones. The similarity of the conditions in the other classrooms within each zone were verified using handheld instruments (TSI Q Track) recording temperature (T), relative humidity (RH), and carbon dioxide (CO_2_). Cleaning policies within each school were also reviewed.

The continuous monitoring lasted an average of 12 weeks both before and after renovation, recording T, RH, and CO_2_ with 10-minute interval as described by Clinchard et al. (2024) [13]. These data were used to assess ventilation adequacy and thermal conditions in the classrooms. In addition, cleaning effectiveness was assessed by taking adenosine triphosphate (ATP) measurements both before and after cleaning of desktops as described by Shaughnessy, et al. (2013) using Charm novaLUM system [19].

Data processing of the continuous monitoring data and assessment of ventilation adequacy is presented by Clinchard et al. (2024) in detail [13]. Hereby, we report the results from combined analyses including ventilation rate (VR) estimated based on daily maximum CO_2_ values as previously described [20] using the CO_2_ generation rates based on Persily and Jonge (2017) [21]. In addition to indoor T and RH data, corresponding outdoor data were retrieved from the closest weather stations using Open-Meteo.com Weather API [22], to estimate both indoor and outdoor absolute humidity (AH) based on Bolton (1980) [23].

### District data and the effects of district wide renovation on illness absence

District data from fall 2016 to spring 2023 included student-level information on absences and socio-demographic variables, such as grade level (K-12), gender, ethnicity, and eligibility for free or reduced-price lunch. Each student’s absences were recorded by the district using a unique student ID, date of absence, and reason for absence (if known). The reporting was based on parent reporting; there was no requirement for associated medical documentation. Full definitions related to absences are presented by Shaughnessy et al. (2022) [14]. This paper focuses on results related to absence due to respiratory and gastrointestinal illness (RI and GI).

First, we studied the effects of renovations and IEQ parameters on student level number of days absent per school year for the given reasons, which were calculated by aggregating data by student id and school year over the seven consecutive school years, during which the students typically moved up one grade by year and some of them changed schools. Second, daily school-level absence rates were calculated as the number of absences per day divided by total enrollment. Descriptive statistics of the dependent variables are presented in Table 1.

**Table 1:** Dependent variables.

	N	Min	Max	Mean	SD
**Student level models**					
Total days absent	138,249	1	171	7.95	7.58
Absence days due respiratory illness	138,249	0	26	0.68	1.58
Absence days due gastrointestinal illness	138,249	0	50	0.59	1.34
Absence days due to asthma	138,249	0	19	0.02	0.27
**School level models**					
% Absent	40,696	0.00	83.00	5.06	3.40
% Absent due respiratory illness	40,696	0.00	15.70	0.52	0.75
% Absent due gastrointestinal illness	40,696	0.00	7.60	0.42	0.48
% Absent due asthma	40,696	0.00	1.60	0.02	0.07

Associations between the dependent variables, renovation status, and measured IEQ parameters were analyzed with IBM SPSS version 31.0.1.0 using General Estimating Equations (GEEs), which estimates population-averaged model parameters and their standard errors. Partial plots were produced using R version 4.3.2. The selection of working correlation structure was based on Corrected Quasi Likelihood under Independence Model Criterion (QICC) in smaller-is-better form. The student level models were fitted with Poisson distribution and log link using an independent correlation structure. School ID * Student ID was used as (repeated) subjects and school year as a within subject variable. The school level models were fitted with normal distribution and identity link using an autocorrelation structure of order 1. School ID was used as (repeated) subjects and date as a within subject variable. Correlated data summary is presented in Table S1.

There was a large difference in reporting of absences following school closure due to COVID-19 in March 2020, which could have been caused by changes in the policies related to illnesses and/or the reporting of absences. This effect was accounted for by including a binary variable indicating absences recorded before and after COVID-19. Sensitivity analyses were conducted to examine effects on a year-by-year basis. Additionally, to reflect variations in data recording, the student-level model included the mean number of days absent per school, while the school-level models included the total absence rate.

Factors and covariates fitted in the models included:

- student’s grade level, gender (female/male), ethnic group (Asian/Hispanic/Multi/White/Other), and eligibility to free lunch (yes/no) according to district recordings (only in student level models)
- school level socio-demographic variables: mean age, % students eligible for free lunch, % Hispanic
- outdoor T, AH, and RH: annual average in student level models and school-day average in school level models
- renovation status (pre/post)
- IEQ variables: estimated VR, T, RH, AH, and pre-cleaning ATP (logarithmic scale, lgATP) pre and post renovation

Categorical variable information based on GEE models is shown in Table 2 and continuous variable information in Table 3. To study whether IEQ variables could explain the effect of renovation, first models (A) included all except IEQ variables, and estimated the overall association between renovations and illness absence. Models B added IEQ variables to assess whether the association between renovations and absenteeism changed in magnitude after accounting for IEQ, which would suggest a potential role for IEQ in this relationship. Models B were also run excluding data from the 2019–2020 and 2020–2021 school years to address potential effects of COVID-19.

**Table 2:** Categorical variable information.

		Student level N (%)	School level N (%)
Renovation status (before renovation)		37,152 (26.9)	13,715 (33.7)
COVID-19 (before March 2020)		73,147 (52.9)	20,916 (51.4)
Female		67,144 (48.6)	
Eligible for free lunch		31,938 (23.1)	
Ethnic group	Asian	6563 (4.7)	
	Hispanic	27,815 (20.1)	
	Multi	8214 (5.9)	
	Other	1865 (1.3)	
	White	93,792 (67.8)

**Table 3:** Continuous variable information.

Covariates	N	Min	Max	Mean	SD
**Student level models**					
Grade level	138,249	PK	12	6.12	3.99
Age, years	138,249	7.80	17.70	12.27	3.46
% free lunch	138,249	0.92	77.08	23.14	14.69
% Hispanic	138,249	2.46	72.78	20.12	14.92
Outdoor T	138,249	4.18	12.58	10.72	1.15
Outdoor RH	138,249	41.08	54.14	46.37	2.91
Outdoor AH	138,249	3.30	5.17	4.68	0.28
VR	138,249	1.38	15.45	5.30	3.18
Indoor T	138,249	20.10	23.53	21.54	0.70
Indoor RH	138,249	14.93	37.37	26.05	4.61
Indoor AH	138,249	2.87	6.91	4.93	0.88
LgATP	138,249	4.38	6.07	5.25	0.32
**School level models**					
Mean age, years	40,696	7.80	17.70	10.79	3.01
% Free lunch	40,696	0.92	77.08	24.27	16.65
% Female	40,696	31.93	56.00	48.53	3.01
% Asian	40,696	0.00	24.11	4.53	3.89
% Hispanic	40,696	2.46	72.78	20.03	17.11
% Multi	40,696	1.60	15.02	6.16	2.18
% Other	40,696	0.00	5.93	1.31	0.79
% White	40,696	24.11	90.63	67.97	15.80
Outdoor T	40,696	-24.78	32.97	8.87	10.54
Outdoor RH	40,696	8.17	99.83	46.93	19.43
Outdoor AH	40,696	0.53	12.93	4.17	2.19
VR	40,696	1.38	15.45	4.58	2.90
Indoor T	40,696	20.10	23.53	21.53	0.70
Indoor RH	40,696	14.93	37.37	26.04	4.89
Indoor AH	40,696	2.87	6.91	4.99	0.92
LgATP	40,696	4.38	6.07	5.28	0.32

## Results

On average, students missed eight days of school per year, with 9% due to RI and 7% to GI (see Table 1). About 31% of absences were reportedly due to other illnesses, while 54% had no given reason, some of which were likely related to RI and GI. The average daily absence rate was 5%, with approximately 10% due to RI and 8% to GI.

School level average IEQ variables both before and after renovation are shown in Table S2. Estimated mean ventilation rates increased post-renovation. Indoor T remained similar but had a smaller standard deviation. Both average RH and AH decreased. Pre-cleaning ATP levels also decreased, while post-cleaning ATP levels remained unchanged.

About one third of the district data were collected before renovations, and slightly over 50% were collected before COVID-19 related school closures. The student population was 49% female, and the average age, based on the grade level, was 12. Some 23% were eligible for free lunch. Two thirds were White and about 20% Hispanic, whereas the other ethnic groups comprised about 12% of the population. All these student level background variables were significantly associated with the number of days absent per school year and included in the student level GEE models. On the school level, background variables included mean days absent per school year, mean age (reflecting school level, i.e. corresponding to elementary, middle and high school), % of students eligible for free lunch, % Hispanic, outdoor T, and COVID-19 (pre/post). These variables were commonly significantly associated with the number of days absent per school year variables and included in all models. After including % Hispanic, adding the other school level ethnic composition variables did not improve the model fit, hence excluded from the models.

**Fig. 1:**
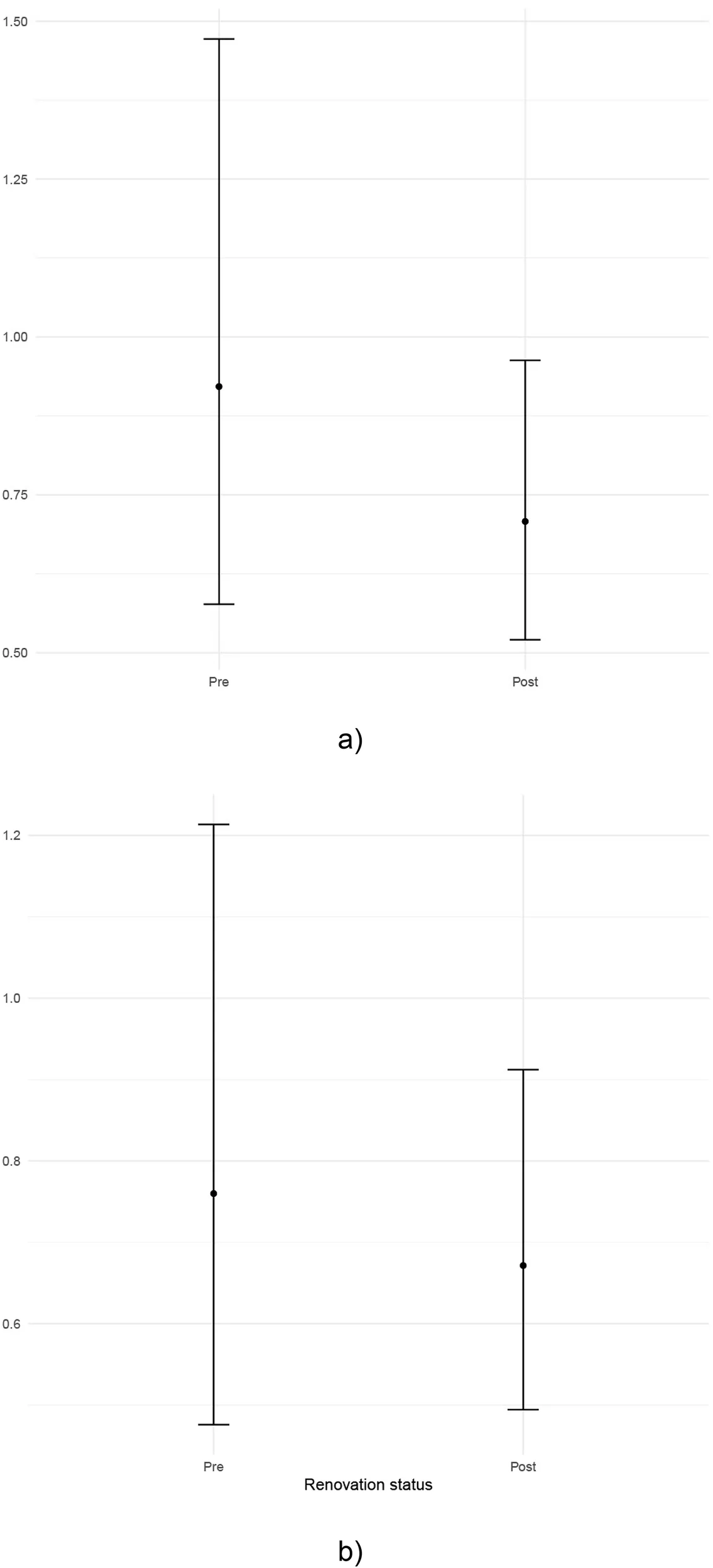
Predicted counts of absence before and after renovations. **a** shows respiratory illness (RI), and **b** gastrointestinal illness (GI).

Based on Model A, renovation status was significantly (*p* < 0.001) associated with the number of days absent due to RI (IRR1.34, 95% CI 1.27–1.40), where the number of days absent was higher before renovations (see Fig. 1 and Table S3). Number of days absent due to RI were reported significantly lower before COVID-19 school closures in both models. When school years 2019–2020 and 2020–2021 were excluded, the effect of renovation became stronger (IRR1.80, 95%CI 1.68–1.93).

**Fig. 2:**
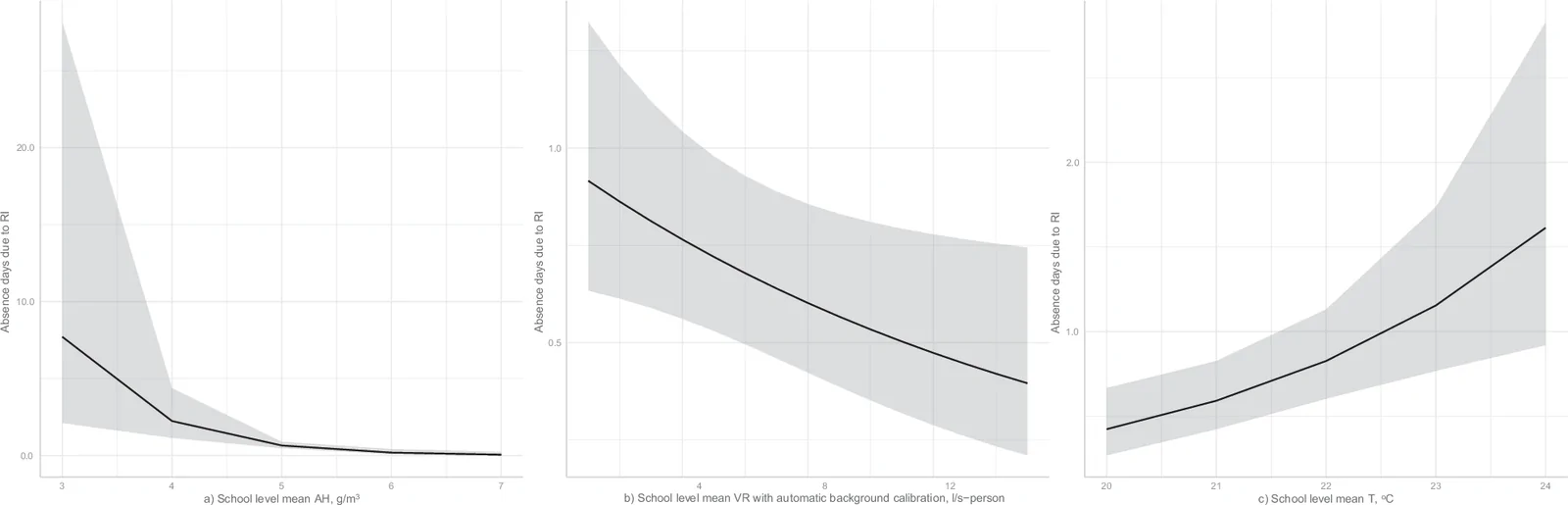
Partial plots of the associations between respiratory illness (RI) and indoor environmental variables. **a** shows the association with absolute humidity (AH), **b** ventilation rate (VR), **c** temperature (T).

Based on Model B, inclusion of IEQ variables resulted in a small difference in the estimated association between days absent due to RI and renovations. In this model, lower indoor AH, VR, and higher indoor T associated (*p* < 0.001) with higher number of days absent (see Fig. 2), and the association with higher lgATP was nearly significant (*p* < 0.1). Higher indoor RH appeared to associate with higher number of days absent due to RI (IRR 1.22, 95%CI 1.12–1.24). However, due to indoor RH being dependent of indoor T and AH, we also tested the model without those variables resulting in an opposite effect (IRR 0.98, 95%CI 0.98–0.98). Excluding RH resulted IRR1.09 (95% CI 1.076–1.11) for indoor T and IRR 0.86 (95%CI 0.84–0.88) for indoor AH.

Similarly, there was a significant association between renovation status and GI (IRR1.25, 95%CI 1.20–1.31) based on Model A (Fig. 1, Table S4). Number of days absent due to GI were reported significantly lower before COVID-19 school closures, but the difference was not as large as with respect to RI. When school years 2019–2020 and 2020–2021 were excluded, the effect of renovation became stronger (IRR 1.37 95%CI 1.29–1.46).

**Fig. 3:**
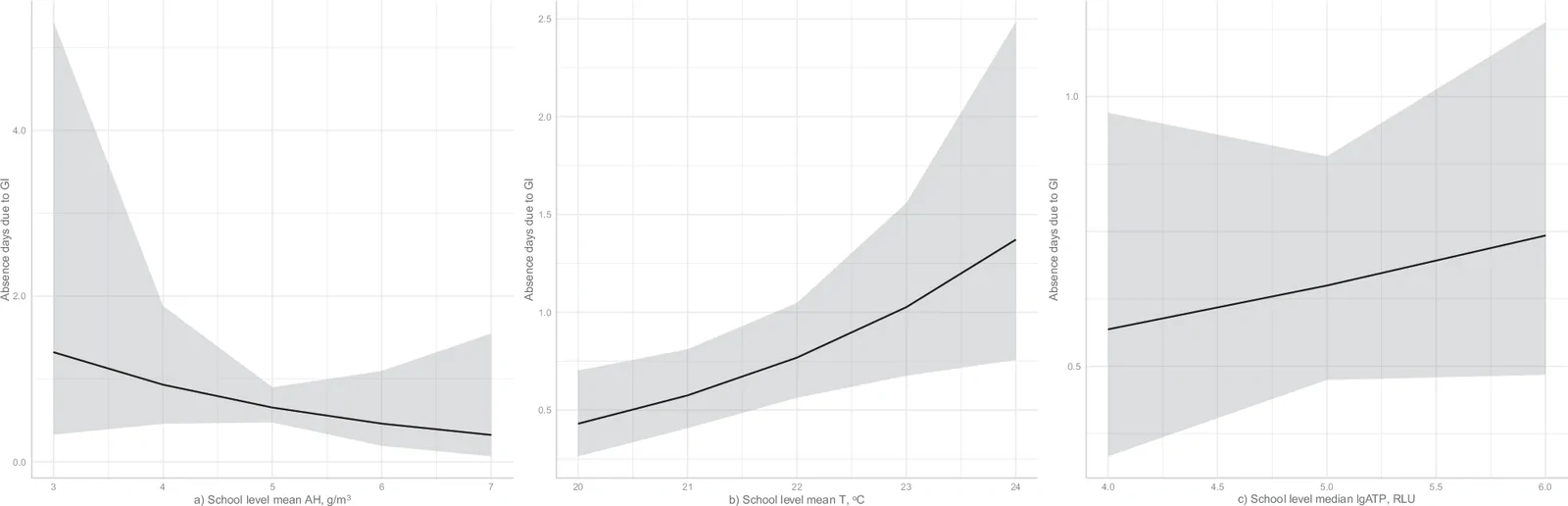
Partial plots of the associations between gastrointestinal illness and indoor environmental variables. **a** shows the association with absolute humidity (AH), **b** temperature (T), and **c** log-transformed ATP (lgATP).

Based on Model B, inclusion of IEQ variables resulted in a modest change in the estimated association between days absent due to GI and renovations. The associations with IEQ parameters were significant for all but VR: higher indoor T and lgATP as well as lower AH corresponded with higher number of days absent (see Fig. 3). Higher indoor RH associated with higher number of days absent (IRR 1.08, 95%CI 1.05–1.10). Excluding indoor T and AH from the model resulted IRR 1.01 (95% CI1.01–1.01), whereas excluding indoor RH resulted in IRR 1.21 (95%CI 1.18–1.23) for indoor T and IRR 1.05 (95%CI 1.03–1.07) for indoor AH, correspondingly.

The total number of days absent associated were significantly lower before renovation (IRR 0.97, 95%CI 0.95–0.99). The results remained closely similar when adding IEQ variables in Models B. In addition, total number of days absent was significantly associated with ventilation rate (IRR 1.00, 95%CI 1.00–1.01), indoor T (IRR 0.98, 95%CI 0.97–1.00), AH (IRR 1.05, 95%CI 1.01–1.10), RH (IRR 0.99, 95%CI 0.98–1.00), and lgATP (IRR 0.97, 95%CI 0.95–1.00). In addition, total number of days absent was significantly higher before COVID-19 school closures. These results are shown in Table S5.

Results related to school level daily absence rates, renovation status and IEQ are shown in Table S6. Based on Models A, renovation status was not significantly associated with any of the dependent variables, although there was a nearly significant association (*p* = 0.053) for gastrointestinal absence rate. Based on Models B, lower VR and AH, and higher indoor T and RH associated with higher respiratory illness absence rate, and higher indoor T associated with higher gastrointestinal illness absence rate. On the other hand, lower lgATP associated with higher total absence rate, with wide confidence intervals. When school years 2019–2020 and 2020-–2021 were excluded, the effect of renovation became statistically significant (*p* = 0.017).

## Discussion

In this study, hierarchical (multilevel) models were used to reflect the nested data structure, with repeated observations within students and students within schools. This approach accounts for  dependencies among observations, enables appropriate partitioning of variance across individual and school levels, and captures unobserved heterogeneity across schools through random effects (e.g., differences in building characteristics and practices). Longitudinal modeling accounted for repeated observations over time and leveraged within-student variation in exposures and outcomes, allowing each student to serve as their own control. This improves statistical efficiency and reduces bias from unmeasured, time-invariant individual characteristics that may influence absenteeism. Longitudinal models are particularly well suited to absenteeism outcomes, which are episodic and vary over time in response to environmental conditions and illness dynamics.

Whereas some previous studies did not have data on specific reasons of absence and therefore studied the associations between ventilation rates and absence due to broader range of illness or medical conditions [24], our study focused specifically on absence due to RI and GI. It was also noticed that results for total absence days or rates are somewhat contradictory. For example, higher ventilation rates associated with more absences, while higher biocontamination levels associated with fewer absences. Total absences include various non-illness-related reasons (e.g., unexcused absences, family events, appointments), which may not accurately reflect the impact of school environment and IEQ on student health. Additionally, school-level differences in recording illness-related absences could not be accounted for in the total absence models.

Student-level data revealed significant associations between the number of days absent and various factors and covariates in the models. In our study, grade level was treated as a continuous variable because it also represents student age. At the school level, we included the mean student age to account for demographic differences between schools (mean age across elementary school grades is 9, middle school 13, and high school 16). It is noteworthy that behavioral patterns change with age: young children tend to remain in one classroom, whereas middle and high school students increasingly rotate between rooms. It is reasonable to assume that this transition occurs gradually across grade levels. Including school type (elementary/middle/high school) as a factor (categorical variable) in the models had only modest effects on parameter estimates and did not alter our conclusions regarding the renovation effect (data not shown).

In the student level models, renovation status associated with both RI and GI and remained significant after IEQ parameters were added, where number of days absent were significantly lower after renovation. Changes in the IRRs indicate that the associations with renovation status were at least partly related to changes in IEQ. In contrast, while school-level daily absence rates could be used to track and even predict absences across school districts, the statistical power was lower, and the associations were weaker.

COVID-19 substantially altered absenteeism patterns. The effects of COVID-19 were examined on a year-by-year basis. While the estimates were comparable across other years, they differed markedly for the 2019–2020 and 2020–2021 school years. This was likely due to school closures beginning in spring 2020 and changes in school operations during the 2020–2021 school year. Operations then returned to normal during the 2021–2022 school year. Excluding data from the 2019–2020 and 2020–2021 school years resulted in stronger associations between renovation and both RI and GI absenteeism in student-level models (Tables S3 and S4). In contrast, associations in school-level models were largely unchanged, except for total absence rate, which reached statistical significance (Table S6). Estimates for IEQ parameters remained comparable across models, supporting the conclusion that the findings are robust and reliable.

Many classrooms had inadequate ventilation before renovations, and in some classrooms, ventilation rates stayed below the recommended minimum even after renovations [13]. Higher VR associated with lower number of days absent due to RI (IRR 0.94) in the student level model and lower absence rate due to RI (IRR 0.98) in the school level model.

Other indicators of ventilation performance, such as peak CO_2_ levels and ACR, were analyzed using a novel methodology to segment build-up and decay periods [13]. However, VR provided better goodness of fit in illness absence models, possibly due to lower variability compared to CO_2_ and the challenges of interpreting ACR relative to space volume. Thus, VR may be a more reliable indicator of ventilation performance in relation to illness absences.

Indoor T was within recommended ranges in most classrooms both pre- and post-renovation. In student level models, higher indoor T was associated with higher number of days absent due to RI (IRR 1.40) and GI (IRR 1.34). In the school level models higher indoor T associated with higher GI absence rate. It appears that within the range of 20 °C–24°C, cooler temperatures could be more beneficial for school health.

It should be noted that that indoor T is not a deterministic function of outdoor T [25]. Mechanical heating, ventilation system operation, classroom occupancy, and building envelope characteristics can all produce substantial divergence from outdoor conditions. Moreover, indoor T interacts with indoor humidity in ways that are directly relevant to pathogen survival and transmission. For these reasons, indoor T provides meaningful exposure information beyond outdoor T alone.

Both RH and AH in the monitored classrooms were lower after the renovations, and it could to some extent be related to higher ventilation rate during the cold season. However, due to low outdoor ambient humidity values during winter, optimal indoor humidity may not be reasonably always achieved. Low RH has been associated with discomfort, irritation symptoms, and potentially increased spread of infectious diseases. Therefore, an intermediate RH (40–60%) has been recommended [26], though no universally accepted health-based guidelines for indoor RH or AH exist.

Based on this study, higher indoor AH (in grams per cubic meter of air), was associated with lower RI and GI. When all IEQ variables were included in the models, higher indoor RH associated with higher number of days absent due to RI and GI, but when indoor T and RH were removed, the association between RH and RI turned negative (higher RH associated with lower number of days absent), and the positive association between RH on GI was diluted. In the school level models, higher AH associated with lower RI absence rate. It appears higher amount of moisture in the air could be protective of health, whereas RH is dependent on both AH and indoor T and may have indirect influence.

Given that RH is dependent on both indoor T and AH, it is difficult to differentiate their effects. However, their relationship is not straight forward: whereas T directly relates to the amount of moisture the atmosphere can hold, AH in relation to that amount determines RH. Indoor AH is impacted by both outdoor AH and indoor generated moisture and moisture removed by air conditioning. Nevertheless, the goodness of fit (based on QICC) was better for models including all three variables, with consistent findings for indoor AH and T.

Peci et al. (2019) reported a negative association between outdoor AH and T and influenza, whereas the effect of outdoor RH was controversial [27]. Also, Wiemken et al. (2017) found outdoor AH negatively associated with hospitalizations due to influenza [28]. Our results were similar for RI, GI, and total absence rates. On the other hand, in the student level models, higher annual average outdoor AH corresponded with higher number of days absent due to RI and GI. Higher annual average AH could be related to, for example, precipitation.

It has been reported that higher RH rather than AH is beneficial for indoor air pathogens’ survival [29, 30]. In our models, where both indoor T and AH were included, higher indoor RH associated with higher number of days absent due to RI and GI, but the effect turned opposite when indoor T and AH were not included. It is possible that the effects of T and/or AH mask the opposite effect of indoor RH, when all three variables are not included (e.g., due to multicollinearity issues when sample sizes are too small).

ATP is a measure of biological contamination, and high correlations have been found between ATP levels on desktops and microbes including bacteria, fungi, and yeast [31], but not viruses [32]. ATP monitoring showed a decrease in pre-cleaning ATP levels after renovation, while post-cleaning levels remained unchanged. These results suggest that cleaning effectiveness improved due to more frequent cleaning, but the cleaning methods themselves were not more effective. Higher pre-cleaning lgATP associated significantly with higher number of days absent due to GI (IRR 1.15), but the association with RI (IRR1.06) did not reach statistical significance (*p* < 0.10). We could speculate that the stronger associations with GI suggest it may be more easily transmitted through inanimate objects.

To summarize, our results indicate that more than one-third of absences related to RI and GI could be reduced by upgrading school facilities and following recommended standards for ventilation and surface cleanliness. Indoor environmental parameters represent distinct, proximal exposures that may mediate renovation effects but also remain important to model for interpretation and policy relevance. For example, a linear reduction in RI was observed with VR increasing from 2 to 13 L/s-person, while GI was reduced with ATP levels decreasing from 10⁶ to 10⁴ RLUs. Further reductions could be achieved by maintaining higher humidity and cooler temperatures in classrooms, but adjusting these factors is more complex as they are influenced by outdoor climate. Nevertheless, consistent reductions in both RI and GI were observed with AH increasing from 3 to 7 g/m³ and T decreasing from 24 °C to 20 °C. We also acknowledge that future work could apply formal causal mediation methods to quantify indirect effects more rigorously.

Overall, this study conducted IEQ monitoring before and after renovations, with feedback provided to facility management. Improvements were observed in ventilation rates, biocontamination levels on high-touch surfaces, and more consistent indoor temperatures. The findings highlight the importance of maintaining good IEQ in schools to promote student health and reduce absences due to infectious diseases.

## Supplementary information

Supplementary information

## Data Availability

The restrictions prohibiting the authors from making the data set publicly available are due to part of the data being obtained from a school district. The readers may contact the corresponding author for data requests.
