# Supplementary material for: School district-wide renovations, indoor environmental quality, and illness absence
Source: J Expo Sci Environ Epidemiol. 2026 May 6;36(6):1221–8. doi: 10.1038/s41370-026-00903-5 (PMC13727683; doi:10.1038/s41370-026-00903-5)
Supplement: Supplementary file 1 [file 41370_2026_903_MOESM1_ESM.docx]

**Glossary of Abbreviations**

AH (Absolute Humidity): Mass of water vapor per unit volume of air.

API (Application Programming Interface): Software interface for accessing external data.

ATP (Adenosine Triphosphate): Surface-based indicator of biological residue and cleanliness.

Beta coefficient (β): A parameter estimate from a regression model that represents the change in the outcome variable associated with a one-unit increase in a predictor variable, holding other variables constant.

CO₂ (Carbon Dioxide): Indicator of ventilation adequacy.

COVID-19 (Coronavirus Disease 2019): Respiratory illness caused by SARS-CoV-2.

GEE (Generalized Estimating Equations): Method for analyzing correlated data.

GI (Gastrointestinal Illness): Illness affecting the digestive tract.

HVAC (Heating, Ventilation, and Air Conditioning): Systems controlling indoor air and thermal conditions.

ID (Identification code): Unique identifier used to distinguish individuals or records.

IEQ (Indoor Environmental Quality): Conditions of the indoor environment affecting health and comfort.

IRR (incidence rate ratio): A measure of association that compares the rate of an outcome (e.g., days absent) between exposure groups over time.

K–12 (Kindergarten through Grade 12): Primary and secondary education levels.

QICC (Quasi-likelihood under the Independence Model Criterion): Model selection criterion for GEE.

RH (Relative Humidity): Percentage of water vapor in air relative to saturation.

RI (Respiratory Illness): Illness affecting the respiratory tract.

T (Temperature): Outdoor or indoor air temperature.

VR (Ventilation Rate): Rate of air supplied to a space.

Table S1. Correlated data summary.

| Number of Levels | Outcome type | Days absent | Absence rate |
| --- | --- | --- | --- |
|  | Subject Effect |  |  |
|  | School ID | 45 | 45 |
|  | Student ID | 45428 |  |
|  | Within-Subject Effect |  |  |
|  | Date |  | 1217 |
|  | School year | 7 |  |
| Subjects | |  | 45 |
| Measurements per Subject | Minimum | 1 | 629 |
|  | Maximum | 7 | 1064 |

Table S2. Descriptive statistics of school level average IEQ variables before and after renovation.

|  | Before renovation | After renovation |
| --- | --- | --- |
|  | Mean (Min-Max), SD | Mean (Min-Max) SD |
| Ventilation rate [L/s-person] | 3.15 (1.38-8.86), 1.61 | 5.15 (2.10-15.45), 2.89 |
| Indoor T [oC] | 21.69 (20.10-23.53), 0.81 | 21.59 (20.47-22.80), 0.59 |
| Indoor RH [%] | 29.92 (23.03-38.63), 4.15 | 24.61 (14.93-36.30), 5.03 |
| Indoor AH [g/m3] | 5.76 (4.50-7.6 0) 0.76 | 4.71 (2.87-7.21), 0.96 |
| Log (ATP*) pre-cleaning [*RLUs] | 5.55 (4.38-6.07), 0.30 | 5.22 (4.50-5.72), 0.25 |
| Log (ATP*) post-cleaning [* RLUs] | 4.33 (3.46-4.90), 0.27 | 4.33 (3.15-4.92), 0.47 |

Table S3. Associations between days absent due to RI, renovation status, and IEQ.

|  | Model A | Model B | Model B^c^ |
| --- | --- | --- | --- |
|  | IRR (95%CI) | IRR (95%CI) | IRR (95%CI) |
| Intercept | 23.028 (9.983-53.120) *** | 0.098 (0.032-0.303) *** | 147.805 (42.512-513.883) *** |
| Student level variables |  |  |  |
| Gender, female | 0.949 (0.923-0.977) *** | 0.947 (0.921-0.974) *** | 0.939 (0.912-0.966) *** |
| Gender, male ^b^ | 1 | 1 | 1 |
| Ethnicity |  |  |  |
| Asian | 0.622 (0.571-0.678) *** | 0.638 (0.586-0.695) *** | 0.641 (0.586-0.701) *** |
| Hispanic | 0.832 (0.797-0.868) *** | 0.833 (0.797-0.869) *** | 0.834 (0.797-0.873) *** |
| Multi | 0.964 (0.909-1.022) | 0.966 (0.912-1.024) | 0.953 (0.898-1.012) |
| Other | 0.618 (0.534-0.715) *** | 0.637 (0.550-0.737) *** | 0.636 (0.549-0.738) *** |
| White ^b^ | 1 | 1 | 1 |
| Not eligible for free lunch | 0.990 (0.953-1.029) | 0.991 (0.954-1.030) | 1.004 (0.964-1.045) |
| Eligible for free lunch ^b^ | 1 | 1 | 1 |
| Grade level | 0.951 (0.945-0.957) *** | 0.951 (0.945-0.957) *** | 0.954 (0.947-0.961) *** |
| School level variables |  |  |  |
| Mean days absent per school year | 1.099 (1.093-1.106) *** | 1.094 (1.088-1.101) *** | 0.996 (0.988-1.004) |
| Mean age ^a^ | 0.945 (0.937-0.953) *** | 0.955 (0.946-0.963) *** | 0.998 (0.988-1.007) |
| % Free lunch | 1.001 (0.998-1.003) | 1.003 (1.001-1.005) * | 1.006 (1.003-1.008) *** |
| % Hispanic | 0.994 (0.992-0.997) *** | 0.994 (0.992-0.997) *** | 0.994 (0.992-0.997) *** |
| Annual average outdoor T | 0.645 (0.619-0.672) *** | 0.619 (0.594-0.646) *** | 0.508 (0.485-0.533) *** |
| Annual average outdoor AH | 5.454 (4.884-6.089) *** | 6.340 (5.672-7.087) *** | 9.522 (8.381-10.818) *** |
| Annual average outdoor RH | 0.873 (0.857-0.889) *** | 0.850 ( 0.834-0.866) *** | 0.767 (0.750-0.784) *** |
| Before COVID-19 | 0.582 (0.554-0.612) *** | 0.650 (0.618-0.683) *** | 0.413 (0.387-0.441) *** |
| After COVID-19 ^b^ | 1 | 1 | 1 |
| Before renovation | 1.337 (1.272-1.404) *** | 1.302 (1.235-1.372) *** | 1.803 (1.681-1.934) *** |
| After renovation ^b^ | 1 | 1 | 1 |
| Ventilation rate [l/s-person] |  | 0.942 (0.934-0.950) *** | 0.940 (0.932-0.948)*** |
| Indoor T [^o^C] |  | 1.397 (1.353-1.442) *** | 1.293 (1.25-1.338) *** |
| Indoor AH [g/m3] |  | 0.291 (0.262-0.322) *** | 0.379 (0.339-0.424) *** |
| Indoor RH [%] |  | 1.218 (1.196-1.241) *** | 1.159 (1.137-1.183) *** |
| LgATP pre [RLU] |  | 1.055 (0.993-1.120) + | 0.986 (0.926-1.050) |

^a^ Grade weighted average

^b^ Set to zero because this parameter is redundant.

^c^ School years 2019-2021 excluded

***p≤0.001

**p≤0.01

*p≤0.05

+p≤0.1

Table S4. Associations between days absent due to GI, renovation status, and IEQ.

|  | Model A | Model B | Model B^c^ |
| --- | --- | --- | --- |
|  | IRR (95%CI) | IRR (95%CI) | IRR (95%CI) |
| Intercept | 2.664 (1.161-6.115) * | <0.004 (0.001-0.013) *** | 0.183 (0.048-0.696) * |
| Student level variables |  |  |  |
| Gender, female | 0.969 (0.942-0.997) * | 0.967 (0.940-0.994) * | 0.960 (0.932-0.989) ** |
| Gender, male ^b^ | 1 | 1 | 1 |
| Ethnicity |  |  |  |
| Asian | 0.481 ( 0.445-0.521) *** | 0.487 (0.450-0.527) *** | 0.495 (0.455-0.538) ** |
| Hispanic | 0.933 (0.895-0.974) ** | 0.935 (0.896-0.975) ** | 0.946 (0.905-0.989) * |
| Multi | 0.900 (0.847-0.957) *** | 0.903 (0.850-0.960) *** | 0.898 (0.842-0.958) *** |
| Other | 0.804 (0.701-0.922) ** | 0.810 (0.706-0.929) ** | 0.7979 (0.694-0.919) ** |
| White ^b^ | 1 | 1 | 1 |
| Not eligible for free lunch | 0.819 (0.789-0.850) *** | 0.819 (0.789-0.850) *** | 0.831 (0.799-0.864) *** |
| Eligible for free lunch ^b^ | 1 | 1 | 1 |
| Grade level | 1.003 (0.997-1.009) | 1.004 (0.998-1.010) | 1.004 (0.997-1.010) |
| School level variables |  |  |  |
| Mean days absent per school year | 1.108 (1.101-1.115) *** | 1.102 (1.095-1.110) *** | 1.041 (1.032-1.050) *** |
| Mean age ^a^ | 0.936 (0.929-0.944) *** | 0.943 (0.935-0.952) *** | 0.973 (0.964-0.983) *** |
| % Free lunch | 1.005 (1.002-1.007) *** | 1.004 (1.002-1.007) ** | 1.005 (1.003-1.008) *** |
| % Hispanic | 0.994 (0.991-0.996) *** | 0.994 (0.991-0.996) *** | 0.995 (0.993-0.997) *** |
| Annual average outdoor T [^o^C] | 0.849 (0.814-0.885) *** | 0.819 (0.784-0.854) *** | 0.759 (0.723-0.789) *** |
| Annual average outdoor AH [g/m3] | 2.151 (1.917-2.413) *** | 2.412 (2.150-2.705) *** | 2.693 (2.356-3.077) *** |
| Annual average outdoor RH [%] | 0.933 (0.916-0.950) *** | 0.917 (0.900-0.934) *** | 0.882 (0.863-0.902) *** |
| Before COVID-19 | 0.896 (0.853-0.941) *** | 0.920 (0.876-0.966) *** | 0.702 (0.661-0.746) *** |
| After COVID-19 ^b^ | 1 | 1 | 1 |
| Before renovation | 1.251 (1.195-1.309) *** | 1.132 (1.077-1.189) *** | 1.374 (1.291-1.463) *** |
| After renovation ^b^ | 1 | 1 | 1 |
| Ventilation rate [l/s-person] |  | 0.997 (0.989-1.005) | 0.991 (0.982-0.999) * |
| Indoor T [^o^C] |  | 1.336 (1.290-1.384) *** | 1.260 (1.213-1.309) *** |
| Indoor AH [g/m3] |  | 0.704 (0.630-0.787) *** | 0.836 (0.739-0.944) ** |
| Indoor RH [%] |  | 1.076 (1.054-1.098) *** | 1.038 (1.016-1.062) *** |
| LgATP pre [RLU] |  | 1.143 (1.079-1.210) *** | 1.101 (1.037-1.168) ** |

^a^ Grade weighted average

^b^ Set to zero because this parameter is redundant.

^c^ School years 2019-2021 excluded

***p≤0.001

**p≤0.01

*p≤0.05

+p≤0.1

Table S5. Association between total student level absence days, renovation status, and IEQ.

|  | Model A | Model B | Model B^c^ |
| --- | --- | --- | --- |
|  | IRR (95%CI) | IRR (95%CI) | IRR (95%CI) |
| Intercept | 2.681 (1.930-3.724) *** | 4.151 (2.639-6.530) *** | 11.326 (6.527-19.652) *** |
| Student level variables |  |  |  |
| Gender, female | 1.069 (1.056-1.082) *** | 1.069 (1.057-1.082) *** | 1.066 (1.053-1.08) *** |
| Gender, male ^b^ | 1 | 1 |  |
| Ethnicity |  |  |  |
| Asian | 0.740 ( 0.715-0.765) *** | 0.738 (0.713-0.763) *** | 0.735 (0.711-0.759) *** |
| Hispanic | 0.961 (0.943-0.978) *** | 0.961 ( 0.943-0.978) *** | 0.961 (0.942-0.981) *** |
| Multi | 0.974 (0.948-1.000) + | 0.974 (0.948-1.000) + | 0.971 (0.945-0.999) * |
| Other | 0.914 (0.868-0.963) *** | 0.914 (0.867-0.963) *** | 0.907 (0.857-0.961) *** |
| White ^b^ | 1 | 1 | 1 |
| Not eligible for free lunch | 0.866 (0.852-0.880) *** | 0.866 (0.852-0.880) *** | 0.868 (0.853-0.884) *** |
| Eligible for free lunch ^b^ | 1 | 1 | 1 |
| Grade level | 1.011 (1.008-1.014) *** | 1.011 ( 1.008-1.014) *** | 1.011 (1.007-1.014) *** |
| School level variables |  |  |  |
| Mean days absent per school year | 1.134 (1.131-1.137) *** | 1.134 (1.131-1.137) *** | 1.113 (1.109-1.117) *** |
| Mean age ^a^ | 0.985 (0.982-0.989) *** | 0.983 (0.980-0.987) *** | 0.989 (0.985-0.993) *** |
| % Free lunch | 0.999 (0.997-1.000) ** | 0.998 (0.997-0.999) *** | 0.998 (0.997-0.999) ** |
| % Hispanic | 1.000 (0.999-1.015) | 1.000 (0.999-1.001) | 1.000 (0.999-1.001) |
| Annual average outdoor T [^o^C] | 0.999 (0.984-1 .000) + | 1.004 (0.988-1.020) | 0.979 (0.959-0.999) * |
| Annual average outdoor AH [g/m^3^] | 1.030 (0.989-1.073) | 1.021 (0.980-1.064) | 1.066 (1.013-1.121) * |
| Annual average outdoor RH [%] | 1.003 (0.996-1.010) | 1.005 (0.998-1.012) | 0.992 (0.983-1.001) + |
| Before COVID-19 | 1.033 (1.014-1.052) *** | 1.026 (1.007-1.045) *** | 0.965 (0.944-0.987) ** |
| After COVID-19 ^b^ | 1 | 1 | 1 |
| Before renovation | 0.967 (0.949-0.985) *** | 0.983 (0.964-1.002) + | 1.011 (0.988-1.034) |
| After renovation ^b^ | 1 | 1 | 1 |
| Ventilation rate [l/s-person] |  | 1.004 (1.001-1.008) ** | 1.003 (0.999-1.006) |
| Indoor T [^o^C] |  | 0.982 (0.968-0.996) ** | 0.979 (0.964-0.994) ** |
| Indoor AH [g/m^3^] |  | 1.052 (1.007-1.099) * | 1.074 (1.022-1.128) ** |
| Indoor RH [%] |  | 0.990 ( 0.983-0.998) * | 0.986 (0.978-0.995) ** |
| LgATP pre [RLU] |  | 0.973 (0.949-0.997) * | 0.950 (0.926-0.976) *** |

^a^ Grade weighted average

^b^ Set to zero because this parameter is redundant.

^c^ School years 2019-2021 excluded

***p≤0.001

**p≤0.01

*p≤0.05

+p≤0.1

Table S6. Associations between school level daily absence rates, renovation status, and IEQ

|  | Model A | Model B | Model B^c^ |
| --- | --- | --- | --- |
|  | β (95%CI) | β (95%CI) | β (95%CI) |
| **Respiratory illness absence rate** |  |  |  |
| Intercept | 0.997 (0.722-1.273) *** | -2.091 (-4.956-0.775) | -1.580 (-4.684-1.524) |
| Absence rate | 0.063 (0.041-0.084) *** | 0.064 (0.042-0.085) *** | 0.068 (0.047-0.089) *** |
| Mean age ^a^ | -0.056 -(0.079-0.034) *** | -0.046 -(0.067-0.025) *** | -0.049 -(0.074-0.024) *** |
| Free lunch | 0.002 (-0.007-0.011) | 0.003 (-0.005-0.010) | 0.001(0.006-0.009) |
| % Hispanic | -0.005 (-0.017-0.006) | -0.004 (-0.014-0.006) | -0.002(-0.013-0.008) |
| Outdoor T [^o^C] | -0.002 -(0.004-0.001) ** | -0.002 -(0.004-0.001) ** | -0.003 -(0.005-0.001) ** |
| Outdoor AH [g/m^3^] | 0.000 (-0.001-0.000) * | -0.007 -(0.013-0.001) * | -0.007 (-0.016-0.002) |
| Outdoor RH [%] | -0.007 -(0.013-0.001) | 0.000 (-0.001-0.000) | 0.000 (-0.001-0.001) |
| Before COVID-19 | -0.197 -(0.266-0.127) *** | -0.175 -(0.244-0.106) *** | -0.258 -(0.350-0.166) *** |
| After COVID-19 ^b^ | 1 | 1 | 1 |
| Before renovation | 0.017 (-0.083-0.117) | 0.012 (-0.125-0.149) | 0.002 (-0.153-0.157) |
| After renovation ^b^ | 1 | 1 | 1 |
| Ventilation rate [l/s-person] |  | -0.025 -(0.046-0.004) * | -0.025 -(0.048-0.003) * |
| Indoor T [^o^C] |  | 0.138 (0.030-0.245) * | 0.115 (-0.005-0.235) ^+^ |
| Indoor AH [g/m^3^] |  | -0.483 -(0.886-0.080) * | -0.438 (-0.886-0.011) ^+^ |
| Indoor RH [%] |  | 0.071 (0.004-0.137) * | 0.061 (-0.015-0.137) |
| LgATP pre [RLU] |  | 0.121 (-0.128-0.370) | 0.142 (-0.111-0.396) |
| **Gastrointestinal illness absence rate** | Model A | Model B |  |
| Intercept | 0.368 (0.216-0.520) *** | -2.332 -(3.809-0.855) ** | -2.117 -(3.772-0.461) * |
| Absence rate | 0.051 (0.041-0.062) *** | 0.052 (0.042-0.063) *** | 0.050 (0.040-0.061) *** |
| Mean age ^a^ | -0.019 -(0.030-0.008) ** | -0.014 -(0.025-0.003) * | -0.013 -(0.025-0.001) * |
| % Free lunch | 0.004 (-0.000-0.008) + | 0.004 (0.001-0.007) * | 0.003 (-0.000-0.006) ^+^ |
| % Hispanic | -0.003 (-0.007-0.000) + | -0.003 (-0.005-0.000) * | -0.002 (-0.004-0.001) |
| Outdoor T [^o^C] | -0.002 (-0.003-0.000) ** | -0.002 (-0.003-0.000) ** | -0.002 -(0.004-0.001) ** |
| Outdoor AH [g/m^3^] | -0.010 -(0.015-0.006) *** | -0.010 -(0.015-0.006) *** | -0.010 -(0.016-0.005) *** |
| Outdoor RH [%] | 0.000 (-0.001-0.001) | -0.000 (-0.001-0.001) | 0.000 (-0.001-0.001) |
| Before COVID-19 | -0.035 (-0.091-0.020) | -0.034 (-0.084-0.016) | -0.088 -(0.168-0.009) * |
| After COVID-19 ^b^ | 1 | 1 | 1 |
| Before renovation | 0.076 (-0.001-0.154) + | 0.045 (-0.050-0.139) | 0.069 (-0.053-0.191) |
| After renovation ^b^ | 1 |  | 1 |
| Ventilation rate [l/s-person] |  | -0.002 (-0.018-0.014) | -0.002 (-0.021-0.016) |
| Indoor T [^o^C] |  | 0.098 (0.052-0.144) *** | 0.090 (0.032-0.148) ** |
| Indoor AH [g/m^3^] |  | -0.139 (-0.310-0.032) | -0.106 (-0.318-0.105) |
| Indoor RH [%] |  | 0.024 (-0.005-0.054) | 0.017 (-0.020-0.055) |
| LgATP pre [RLU] |  | 0.116 (-0.045-0.276) | 0.117 (-0.050-0.283) |
| **Total absence rate** |  |  |  |
| Intercept | 2.395 (1.052-3.739) *** | 17.117 (1.734-32.501) * | 24.157 (0.881-47.432) * |
| Mean age ^a^ | 0.207 (0.103-0.310) *** | 0.178 (0.053-0.303) ** | 0.227 (0.104-0.351) *** |
| % Free lunch | 0.097 (0.061-0.133) *** | 0.096 (0.060-0.133) *** | 0.073 (0.033-0.113) *** |
| % Hispanic | -0.071 -(0.100-0.043) *** | -0.073 -(0.103-0.044) *** | -0.055 -(0.088-0.021) *** |
| Outdoor T [^o^C] | -0.023 -(0.033-0.012) *** | -0.023 -(0.034-0.012) *** | -0.018 -(0.030-0.007) ** |
| Outdoor AH [g/m^3^] | -0.122 –(0.153-0.090) *** | -0.122 -(0.154-0.091) *** | -0.191 -(0.225-0.158) *** |
| Outdoor RH [%] | 0.003 (0.000-0.006) ^+^ | 0.003 (0.000-0.006) + | 0.004 (0.000-0.008) * |
| Before COVID-19 | 0.044 (-0.478-0.565) | 0.033 (-0.511-0.578) | 0.177 (0.097-0.323) *** |
| After COVID-19 ^b^ | 1 | 1 | 1 |
| Before renovation | 0.013 (-0.615-0.640) | 0.270 (-0.448-0.987) | 1.011 (0.184-1.837) * |
| After renovation ^b^ | 1 | 1 | 1 |
| Ventilation rate [l/s-person] |  | 0.024 (-0.148-0.195) | 0.099 (-0.105-0.303) |
| Indoor T [^o^C] |  | -0.373 (-0.962-0.215) | -0.689 (-1.682-0.304) |
| Indoor AH [g/m^3^] |  | 1.841 (-0.060-3.742) + | 2.863 (-0.195-5.921) ^+^ |
| Indoor RH [%] |  | -0.328 (-0.671-0.015) + | -0.498 (-1.059-0.062) ^+^ |
| LgATP pre [RLU] |  | -1.344 -(2.593-0.094) * | -1.405 -(2.750-0.061) * |

^a^ Grade weighted average ^b^ Set to zero because this parameter is redundant. ^c^ School years 2019-2021 excluded

***p≤0.001	**p≤0.01	*p≤0.05	+p≤0.1
